# Cardiovascular safety and efficacy of metformin-SGLT2i versus metformin-sulfonylureas in type 2 diabetes: systematic review and meta-analysis of randomized controlled trials

**DOI:** 10.1038/s41598-020-80603-8

**Published:** 2021-01-08

**Authors:** Desye Gebrie, Desalegn Getnet, Tsegahun Manyazewal

**Affiliations:** 1 School of Pharmacy, College of Health Sciences, Mekelle University, Mekelle, Ethiopia; 2 Addis Ababa University, College of Health Sciences, Center for Innovative Drug Development and Therapeutic Trials for Africa (CDT-Africa), Addis Ababa, Ethiopia; 3 Pharmacology and Toxicology Course and Research Team, Department of Pharmacy, College of Health Sciences, Adigrat University, Adigrat, Ethiopia

**Keywords:** Cardiology, Endocrinology, Medical research

## Abstract

Diabetes is a serious threat to global health and among the top 10 causes of death, with nearly half a billion people living with it worldwide. Treating patients with diabetes tend to become more challenging due to the progressive nature of the disease. The role and benefits of combination therapies for the management of type 2 diabetes are well-documented, while the comparative safety and efficacy among the different combination options have not been elucidated. We aimed to systematically synthesize the evidence on the comparative cardiovascular safety and efficacy of combination therapy with metformin-sodium-glucose cotransporter-2 inhibitors versus metformin-sulfonylureas in patients with type 2 diabetes. We searched MEDLINE-PubMed, Embase, Cochrane Library, and ClinicalTrials.gov up to 15 August 2019 without restriction in the year of publication. We included randomized controlled trials of patients with type 2 diabetes who were on metformin-sodium-glucose cotransporter-2 inhibitors or metformin-sulphonylureas combination therapy at least for a year. The primary endpoints were all-cause mortality and serious adverse events, and the secondary endpoints were cardiovascular mortality, non-fatal myocardial infarction, non-fatal stroke, hypoglycemia, and changes in glycated hemoglobin A1c (HbA1c), body weight, fasting plasma glucose, blood pressure, high-density lipoprotein cholesterol, and low-density lipoprotein cholesterol. We used a random-effects meta-analysis model to estimate mean differences for continuous outcomes and risk ratio for dichotomous outcomes. We followed PICOS description model for defining eligibility and the Preferred Reporting Items for Systematic Review and Meta-Analysis Protocols (PRISMA-P) 2015 guidelines for reporting results. Of 3,190 citations, we included nine trials involving 10,974 participants. The pooled analysis showed no significant difference in all-cause mortality (risk ration [RR] = 0.93, 95% CI [0.52, 1.67]), serious adverse events (RR = 0.96, 95% CI [0.79, 1.17]) and adverse events (RR = 1.00, 95% CI [0.99, 1.02]) between the two, but in hypoglycemia (RR = 0.13, 95% CI [0.10, 0.17], P < 0.001). Participants taking metformin-sodium glucose cotransporter-2 inhibitors showed a significantly greater reduction in HbA1c (mean difference [MD] = − 0.10%, 95% CI [− 0.17, − 0.03], body weight (MD = − 4.57 kg, 95% CI [− 4.74, − 4.39], systolic blood pressure (MD = − 4.77 mmHg, 95% CI [− 5.39, − 4.16]), diastolic blood pressure (MD = − 2.07 mmHg, 95% CI [− 2.74, − 1.40], and fasting plasma glucose (MD = − 0.55 mmol/L, 95% CI [− 0.69, − 0.41]), p < 0.001. Combination therapy of metformin and sodium-glucose cotransporter-2 inhibitors is a safe and efficacious alternative to combination therapy of metformin and sulphonylureas for patients with type 2 diabetes who are at risk of cardiovascular comorbidity. However, there remains a need for additional long-term randomized controlled trials as available studies are very limited and heterogeneous.

## Introduction

Diabetes mellitus (DM) is one of the top ten causes of death and one of the fastest-growing health problems of the twenty-first century, with 463 million people living with it worldwide in 2019 and this number estimated to be 700 million by 2045^1^. The global direct health expenditure on diabetes mellitus in 2019 was estimated at US$ 760 billion and is expected to increase to a projected US$ 845 billion by 2045^2^. Type 2 diabetes mellitus (T2DM) is the most common and complex form of the disease and accounts for more than 90% of the estimated cases of diabetes, impacting the life expectancy, quality of life, and health of an individual^1,3^. Yet, there is no cure for T2DM, while its prevalence is largely increasing, with increased risk of complications including diabetic retinopathy, neuropathy, kidney damage, and microvascular and cardiovascular complications^4–7^. Cardiovascular disease (CVD) is a common complication and a major cause of morbidity and mortality in patients with T2DM^8,9^.

Despite the introduction of new medications, treating patients with diabetes tend to become more challenging due to the progressive nature of the disease^10–14^. The American Diabetes Association recommends lifestyle interventions (exercise, healthy eating, smoking cessation, and weight reduction) as the first step in treating newly diagnosed T2DM^15^. However, to achieve and maintain specific glycemic targets, the majority of patients require glucose-lowering drugs. Metformin is currently the first-line and widely used pharmacological therapy for patients with T2DM because of its potential benefits, including cardioprotective effect, loss of weight, and prevention of some comorbid diseases^15–22^. If lifestyle interventions and a maximally tolerated dose of metformin fail to achieve the glycemic target within 3 months follow-up, the regimen would be changed to combination therapy^15^.

Metformin-sulfonylurea combination therapy is the most widely used regimen in the management of T2DM^23,24^. Sulfonylureas are prescribed as second-line treatment options in the management of patients with T2DM, while they are still commonly prescribed as a first-line treatment as a substitute to metformin^25^. However, initiating treatment of T2DM with a sulfonylurea in place of metformin is associated with higher rates of ischaemic stroke, cardiovascular mortality, and hypoglycemia^25–31^. On the other side, the use of sulfonylureas as second-line treatments is associated with an increased risk of non-fatal myocardial infarction, all-cause mortality, and severe hypoglycemia, compared with metformin monotherapy; as a result, continuing metformin when introducing sulfonylureas appears to be safer than switching to another drug^32^. Such findings led to new requirements from licensing authorities that all new T2DM therapies should show cardiovascular efficacy and safety^10^.

Sodium-glucose cotransporter-2 inhibitors (SGLT2Is) are novel antidiabetic drugs that can inhibit sodium-glucose cotransporter-2 at the proximal tubule of the kidney. Those novel drugs can decrease renal glucose reabsorption, hyperglycemia, cardiovascular problems and they can increase urinary glucose excretion in patients with T2DM^33–39^. However, there is no clear evidence that shows the relative advantage of either metformin-sulphonylureas or metformin-SGLT2Is combination therapy on major treatment outcomes including CVD^40^. Management of T2DM remains challenging as choosing a second and/or third-line antidiabetic drug is personalized based on efficacy, risk of hypoglycemia, patient's comorbid conditions, impact on weight, side effects, and cost^41^. In particular, although most patients with T2DM require a combination pharmacological therapy, the choice of a best second-line drug is especially critical for the prevention of CVD. Thus, the aim of this systematic review and meta-analysis of randomized controlled trials (RCTs) was to compare the cardiovascular safety and efficacy of combination therapy of metformin-SGLT2Is and metformin-sulfonylureas in patients with T2DM.

## Methods

The protocol for this systematic review and meta-analysis has been registered at the International Prospective Register of Systematic Reviews (PROSPERO) database, ID: CRD42020155616^42^. We followed the Preferred Reporting Items for Systematic Review and Meta-Analysis (PRISMA 2015) guidelines^43^ for the design and reporting of the results.

### Data sources and searches

We searched MEDLINE-PubMed (http://www.ncbi.nlm.nih.gov/pubmed/), Embase (http://www.embase.com/), The Cochrane Library (http://www.cochranelibrary.com/), and ClinicalTtrials.gov (https://www.clinicaltrials.gov/) for completed studies that reported the safety and/or efficacy of metformin-SGLT2Is versus metformin-sulfonylureas combination therapies for patients with T2DM. We included RCTs without restriction on year of publication, but published in English language, up to 15 August 2019. The RCTs were needed to have at least a 1-year follow-up of patients. The keywords we used in different combinations using Boolean Operators were: metformin, biguanide, sodium-glucose co-transporter- 2 inhibitors, SGLT-2 inhibitors, dapagliflozin, canagliflozin, empagliflozin, ertugliflozin, sulfonylurea, gliclazide, glimepiride, glyburide, glibenclamide, glipizide, tolbutamide, type 2 diabetes mellitus, T2DM, cardiovascular outcomes, and randomized controlled trials (Supplementary file 1). All potentially eligible studies were considered for this review, irrespective of the primary outcomes. Manual searching was performed to find out additional eligible trials from the reference lists of key articles.

### Eligibility

We formulated the study’s eligibility criteria using the PICOS (participants, interventions, comparison, outcomes, and study designs) description model^44^:

- Participants
  - Patients with T2DM
  - Man or woman of any age
  - Who was taking a combination therapy of metformin-sulfonylurea or metformin-SGLT2Is
- Intervention
  - A combination of metformin with any of the SGLT2Is, which could be dapagliflozin, canagliflozin, empagliflozin, or ertugliflozin.
- Comparator
  - A combination of metformin with any of sulfonylureas compounds, which could be gliclazide, glipizide, glyburide, glibenclamide, or glimepiride.
- Outcomes
  - Primary endpoints
    - All-cause mortality
    - Serious adverse events (SAEs).
  - Secondary endpoints
    - Cardiovascular mortality
    - Non-fatal myocardial infarction
    - Non-fatal stroke
    - Hypoglycemia
    - Changes in HbA1c
    - Change in body weight,
    - Changes in fasting plasma glucose (FPG)
    - Changes in systolic blood pressure (SBP
    - Changes in diastolic blood pressure (DBP)
    - Changes in low-density lipoprotein cholesterol (LDL-C)
    - Changes in high-density lipoprotein cholesterol (HDL-C)
- Study design
  - RCTs
  - At least one-year duration of follow-up
  - Published in English language

### Study selection

Two independent authors examined the title and abstract of all searched studies. From the title and abstract of all studies identified by the database search, those studies duplicated and not meet the inclusion criteria were excluded. The full texts of the remaining studies were further reviewed. Disagreements were resolved by consensus and if persisted, we arbitrated through discussion with a third author.

### Data extraction

Two independent authors extracted the needed data from each RCTs. These include name of the first author, year of publication, trial registration, mean age of the participant, baseline average body weight, HbA1c, interventions, comparators, number of participants randomized, duration of follow-up, and patient-important outcomes. Data on the mean change of HbA1c (%), body weight (Kg), FPG (mmol/L), SBP (mmHg), DBP (mmHg), HDL-C (mmol/L), and LDL-C (mmol/L) were collected from baseline for continuous outcomes. The status and number of events were captured for the two groups, which include, death, hypoglycemia, adverse events (AEs), SAEs, SAEs related to study drugs, genital mycotic infection (GMI), and adverse cardiovascular events.

### Assessment of risk of bias

We used the Cochrane risk of bias tool^45^ to assess the risk of bias in each included study and the risks were judged by two independent authors as “Low”, “Unclear”, or “High” based on the critical domains, including random sequence generation, allocation concealment, blinding, incomplete outcome data, and selective reporting. Disagreements were resolved by consensus and if persisted, we arbitrated through discussion with a third author.

### Statistical analysis

We carried out meta-analysis using the computer software packages RevMan 5.3^46^ to compare the cardiovascular safety and efficacy between the two combination therapies. Pooled results of continuous patient-important outcomes i.e., HbA1c, FPG, blood pressure, body weight, HDL-C, and LDL-C were reported using a mean difference (MD) with 95% confidence interval (CI). Pooled results of binary outcomes i.e. all-cause mortality, AEs, AEs related to study drug, SAEs, SAEs related to study drug, hypoglycemic event, worsening of coronary artery disease, acute myocardial infarction (AMI), aortic aneurism, coronary artery occlusion (CAO) and GMI were summarized using risk ratio (RR) with 95% CI.

We used Mantel–Haenszel method^47^ to pool effect estimates of dichotomous outcomes and inverse variance for continuous outcomes. The analysis was conducted using a random-effects meta-analyses model as it assumes the observed estimates of the treatment can vary across studies because of the real differences in the treatment effect in each study as well as sampling variability (chance)^48^. We used Cochrane Q test^49^ to assess heterogeneity between studies, and I^2^ testing^50^ to quantify heterogeneity between studies, with values > 50% representing moderate-to-high heterogeneity. We carried out sensitivity and subgroup analysis by duration of the RCTs. We couldn’t conduct funnel plot and Egger test to check any possible reporting bias because the number of studies included in the meta-analyses are insufficient (less than 10 trials). We considered statistical analysis with a p-value < 0.05 statistically significant.

## Results

### Search results

**Figure 1:**
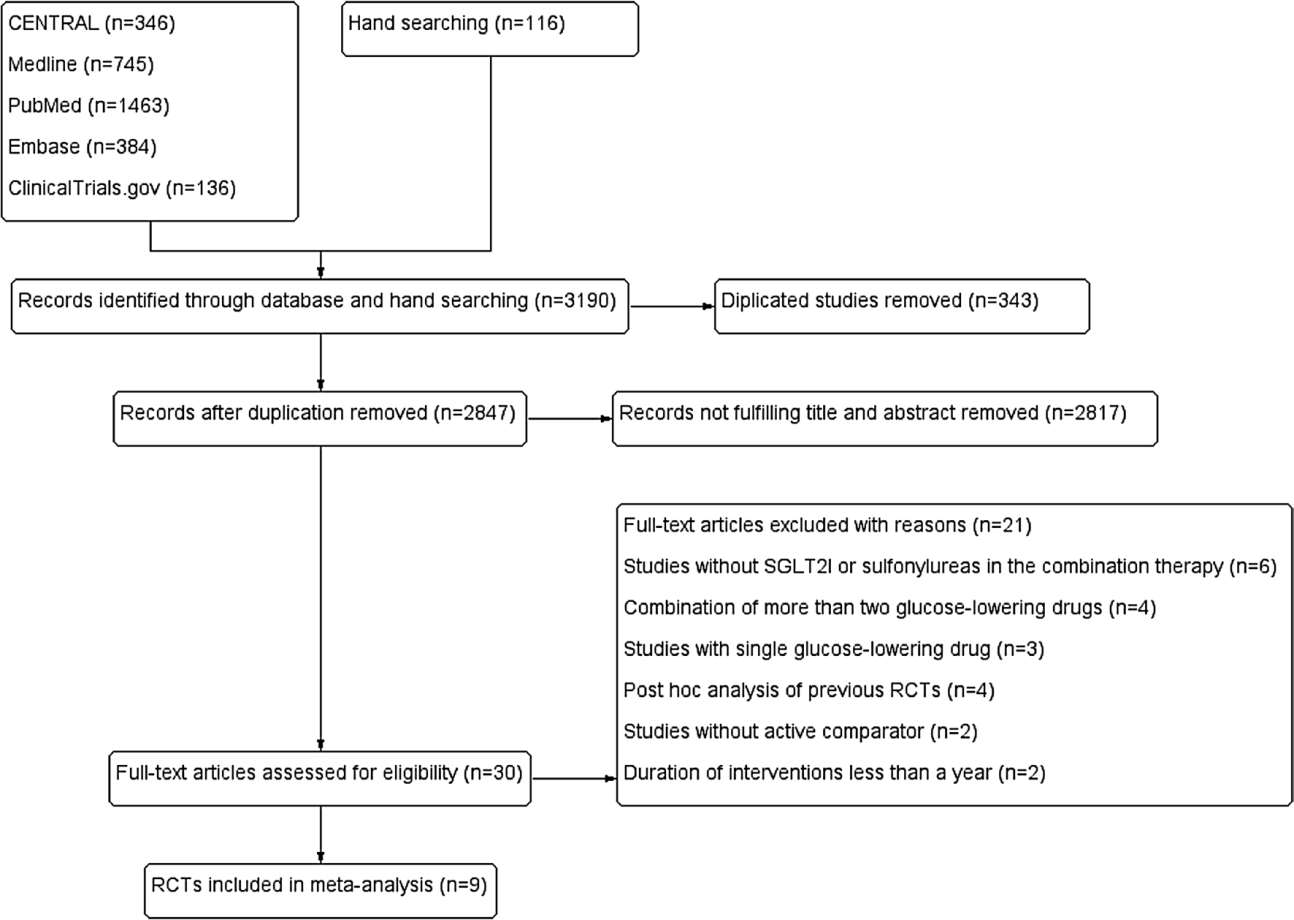
PRISMA flow diagram of the study.

We searched a total of 3,190 citations through the databases, of which we assessed 30 full-text studies for eligibility and found nine of them^51–59^ fulfilled the inclusion criteria. We excluded the rest 21 full-text articles^23,24,60–78^ mainly for they did not include SGLT2Is or sulfonylureas as a combination therapy; included a combination of more than two glucose-lowering drugs; included single glucose-lowering drug; data were driven from review or post hoc analysis of previous RCTs; had no active comparator; or had a duration of interventions less than a year (Fig. 1).

### Study characteristics

Table 1 summarizes the characteristics of the nine RCTs included. Four RCTs^51,54,56,59^ used two different doses of SGLT2Is. The meta-analysis included all results of both doses for dichotomous outcomes but only a high dose of SGLT2Is for continuous outcomes.

**Table 1:** Characteristics of included RCTs.

1st author (year)	Registration	Age (year)	Weight (Kg)	HbA1c (%)	No. of pts	Intervention	Comparator	Follow –up (year)	Patient important outcomes	Met + SGLT2Is	Met + SU
Ridderstråle (2018) ^52^	NCT01167881	≥ 18	82.75	7–10	1545 (Male = 853, female = 692)	Metformin plus Empagliflozin 25 mg (n = 765)	Metformin plus Glimepiride 1–4 mg (n = 780)	4	HbA1c (%)	− 0.29	− 0.10
									weight (Kg)	− 3.08	1.84
									SBP (mmHg)	− 4.1	2.1
									DBP (mmHg)	− 1.8	0.8
									FPG (mmol//L)	− 0.3	0.4
									AEs	706	713
									AEs related to drug	221	277
									SAEs	161	153
									SAEs related to drug	N/A	N/A
									Hypoglycemia	23	218
									Pt received rescue Rx	176	265
									Dyslipidemia	↓fat	↑fat
									GMI	104	30
									CV events	↔	↑
									Death	8	8
Ridderstråle (2014) ^53^	NCT01167881	≥ 18	82.75	7–10	1545 (Male = 853, female = 692)	Metformin plus Empagliflozin 25 mg (n = 765)	Metformin plus Glimepiride 1–4 mg (n = 780)	2	HbA1c	− 0.66	− 0.55
									FPG	− 0.85	− 0.17
									Weight	− 3	1.5
									SBP	− 3.1	2.5
									DBP	− 1.8	0.9
									AEs	661	673
									AEs related to drug	190	252
									SAEs	119	89
									Hypoglycemia	19	189
									Dyslipidemia	41	39
									GMI	131	22
									Pt’s HbA1c < 7%	232	221
									Pt received rescue Rx	113	185
									Death	5	5
Hollander (2019) ^54^	NCT01999218	58.2 ± 9.6	86.9 ± 19.6	7.8 ± 0.6	1315 (Male = 642, female = 673)	Metformin ≥ 1500 mg plus Ertugliflozin 15 mg and Metformin plus Ertugliflozin 5 mg (n = 880)	Metformin ≥ 1500 mg plus Glimepiride 1–8 mg (n = 435)	2	HbA1c	− 0.7	− 0.4
									Weight	− 6.3	1.0
									FPG	− 1.4	− 0.5
									SBP	− 3.2	2.1
									DBP	− 1.5	0.7
									HDL-C	↑	↓
									LDL-C	↔	↔
									Hypoglycemia	35	77
									AEs	622	303
									SAEs	73	30
									GMI	67	3
									SAEs related to drug	4	1
									Pt received rescue Rx	60	31
									Pt’s HbA1c < 7%	228	123
									Death	9	1
Hollander (2017) ^59^	NCT01999218	58.2 ± 9.6	86.8 ± 19.6	7.8 ± 0.6	1325 (Male = 642, female = 683)	Metformin ≥ 1500 mg plus Ertugliflozin 15 mg And Metformin plus Ertugliflozin 5 mg (n = 888)	Metformin ≥ 1500 mg plus Glimepiride 1–8 mg (n = 437)	1	HbA1c	− 1.2	− 0.7
									FPG	− 2.3	− 0.9
									SBP	− 6.0	1.0
									DBP	− 2.1	0.3
									Weight	− 6.4	0.9
									Sever hypoglycemia	2	10
									AEs	525	269
									SAEs	45	12
									Pt received rescue Rx	41	14
									SAE related to drug	3	1
									GMI	56	3
									HDL	↑	↓
									LDL	↑	↓
									Atherosclerosis	0	1
									History of PVD	1	0
									Pt’s HbA1c < 7%	321	190
									Death	6	1
Del Prato (2015) ^58^	NCT00660907	58.2	89.7	7.5	814	Metformin 1500–2500 mg plus Dapagliflozin 2.5–10 mg (n = 406)	Metformin 1500–2500 mg plus Glipizide 5–20 mg (n = 408)	4	HbA1c (%)	− 0.10	0.20
									FPG (mg/dl)	− 13.3	− 3.8
									Weight (Kg)	− 3.65	0.73
									SBP	− 3.69	− 0.02
									DBP	N/A	N/A
									HDL-C	N/A	N/A
									LDL-C	N/A	N/A
									AEs	356	355
									AEs related to drug	127	127
									SAEs	75	81
									SAEs related to drug	9	7
									Hypoglycemia	4	46
									Poor glycemic control	156	182
									Cardiovascular events	0	0
									GMI	5	3
									Death	2	5
Nauck (2014) ^57^	NCT00660907	58.4	88	7.7	814	Metformin ≥ 1500 mg plus Dapagliflozin 2.5–10 mg (n = 406)	Metformin ≥ 1500 mg plus Glipizide 5–20 mg (n = 408)	2	HbA1c	− 0.32	− 0.14
									FPG	− 1.12	− 0.68
									Weight	− 3.7	1.4
									SPB	− 2.7	1.2
									DBP	N/A	N/A
									HDL-C	N/A	N/A
									LDL-C	N/A	N/A
									AEs	337	338
									AEs related to drug	122	118
									SAEs	51	62
									SAEs related to drug	8	7
									Hypoglycemia	17	187
									Aortic aneurysm	0	1
									AMI	1	1
									CAO	0	1
									Ventricular arrhythmia	1	0
									Worsening CAD	1	0
									GMI	60	12
									Poor glycemic control	55	74
									Death	0	4
Nauck (2011) ^55^	NCT00660907	58.4	88	7.7	814	Metformin 1500–2500 mg plus Dapagliflozin2.5–10 mg (n = 406)	Metformin1500–2500 plus Glipizide 5–20 mg (n = 408)	1	HbA1c (%)	− 0.52	− 0.52
									FPG (mmol/L)	− 1.12	− 1.59
									Weight (Kg)	− 3.2	1.2
									SBP (mmHg)	− 3.8	0.9
									DBP (mmHg)	↓	↑
									HDL-C	↑	↓
									AEs	318	318
									AEs related to drug	110	110
									SAEs	35	46
									SAEs related to drug	6	4
									Hypoglycemia	14	162
									GMI	50	11
									Poor glycemic control	1	15
									Acute MI	0	1
									Worsening CAD	1	0
									Death	0	3
Leiter (2015) ^56^	NCT00968812	56.2	86.6	7.8	1450	Metformin plus Canagliflozin 100 mg and Metformin plus canaglifloxin 300 mg (n = 968)	Metformin plus Glimepiride 1–8 mg (n = 482)	2	HbA1c (%)	− 1.39	− 0.55
									FPG (mmol/L)	− 2.4	− 0.6
									Weight (Kg)	− 7.2	0.8
									SBP	− 5.1	1.7
									DBP	− 3.5	− 0.02
									LDL-C (mmol/L)	0.38	0.06
									HDL-C	0.21	0.00
									Triglycerides	↑	↔
									Pt’s HbA1c < 7%	448	212
									AEs	732	378
									AEs related to drug	297	134
									SAEs	94	69
									Hypoglycemia	73	197
									GMI	116	11
									Death	6	2
Cefalu (2013) ^51^	NCT00968812	56.2	86.6	7.8	1450	Metformin plus Canagliflozin 100 mg and Metformin plus canaglifloxin 300 mg (n = 968)	Metformin plus Glimepiride 1–8 mg (n = 482)	1	HbA1c	− 1.75	− 0.81
									FPG	− 2.87	− 1.02
									Weight	− 7.7	0.7
									SBP	− 7.9	0.2
									DBP	− 4.3	− 0.1
									LDL-C	0.37	0.05
									HDL-C	0.18	− 0.01
									Triglycerides	− 0.32	− 0.01
									Pt’s HbA1c < 7%	541	264
									AEs	643	330
									AEs related to drug	263	113
									SAEs	50	39
									Hypoglycemia	51	165
									GMI	97	8
									Death	2	2

*AEs* adverse events, *AMI* acute myocardial infraction, *CAD* coronary artery disease, *CAO* coronary artery occlusion, *CV* cardiovascular, *HbA1c* hemoglobin A1c, *FPG* fasting plasma glucose, *SBP* systolic blood pressure, *DBP* diastolic blood pressure, *HDL-C* high-density lipoprotein cholesterol, *LDL-C* low-density lipoprotein cholesterol, *SAEs* serious adverse events, *GMI* genital mycotic infection, *MI* myocardial infraction, *Rx* treatment, *SGLT2Is* sodium glucose co-transporter 2 inhibitors, *SU* sulfonylurea, *Met* metformin, *N/A* not available.

### Participant characteristics

From the nine RCTs, we polled and included 10,974 patients with T2DM who were in either of the two combination therapies at least for a year (Table 1).

### Methodological quality and risk of bias

**Figure 2:**
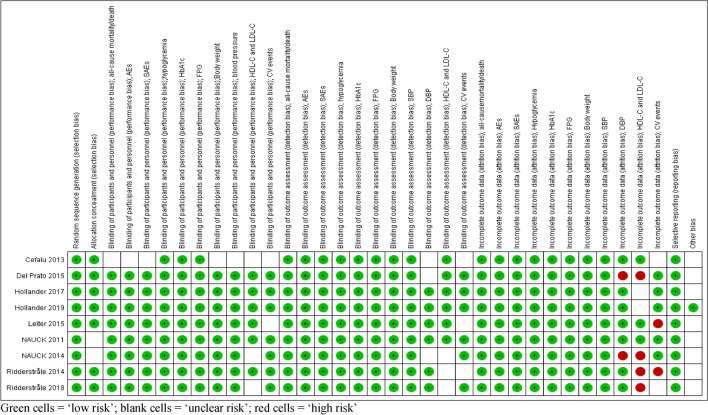
'Risk of bias' summary: review authors' judgments about each 'risk of bias' item for included trials. Green cells = ‘low risk’; blank cells = ‘unclear risk’; red cells = ‘high risk’.

The studies were found to be “low risk of bias” when these studies were subjected to the Cochrane Collaboration’s Tool for Quality Assessment of Randomized Controlled Trials (Fig. 2).

### Efficacy and safety assessments

#### All-cause mortality

**Figure 3:**
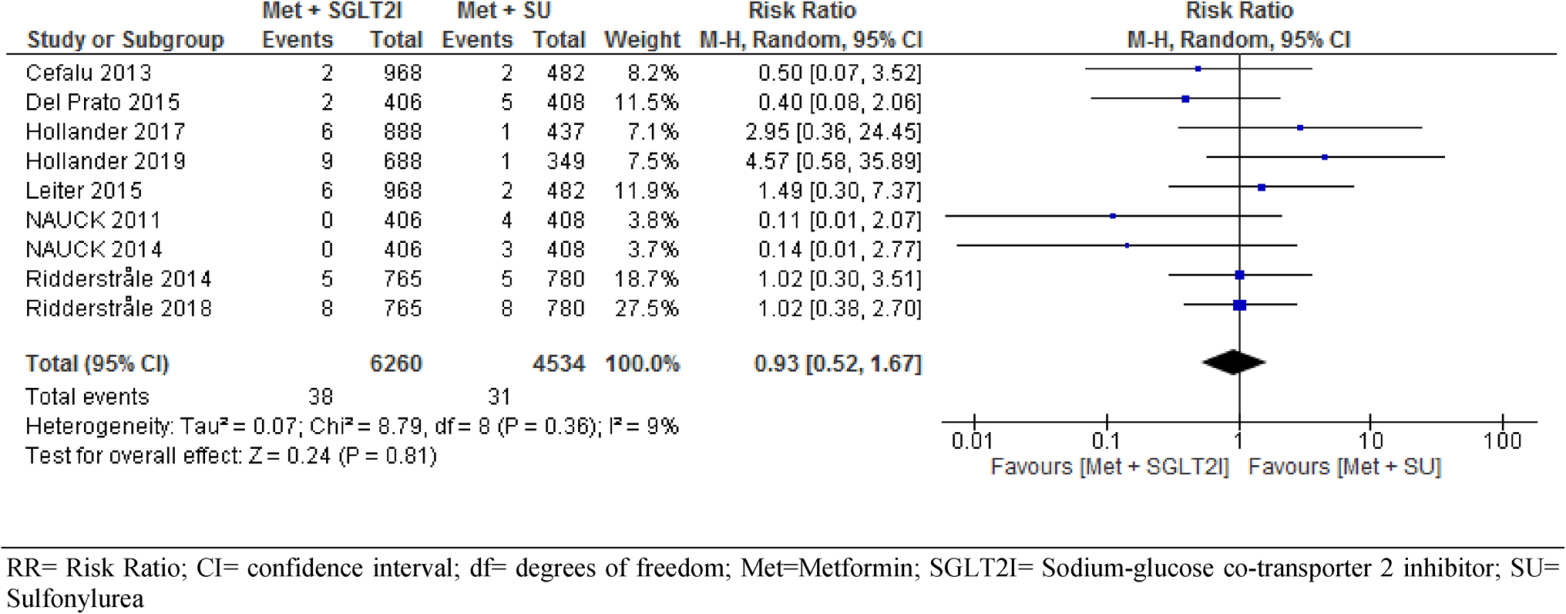
Comparison of all-cause mortality/death events between patients with T2DM who were on metformin-SGIT2I and metformin-sulphonylureas combination therapies. *RR* risk ratio; *CI* confidence interval; *df* degrees of freedom; *Met* metformin; *SGLT2I* sodium-glucose co-transporter 2 inhibitor; *SU* sulfonylurea.

All the nine included RCTs involving 10,974 participants assessed all-cause mortality/death events between intervention and control groups. Our meta-analysis of pooled results revealed no significant difference in all-cause mortality/death events between patients with T2DM who were on metformin-SGIT2Is and metformin-sulphonylureas combination therapies (RR = 0.93, 95% CI [0.52, 1.67], *p* = 0.81), with statistically non-significant heterogeneity between studies (I^2^ = 9%) (Fig. 3). Subgroup analysis by duration of follow-up showed no significant difference in risk of death between the two groups, year 1 (RR = 0.66, 95% CI [0.12, 3.71], p = 0.64, year 2 (RR = 1.25, 95% CI [0.47, 3.32], p = 0.66, and year 4 (RR = 0.80, 95% CI [0.35, 1.84], p = 0.60) (S2).

#### Cardiovascular events

Two trials^55,57^ evaluated the cardiovascular efficacy of metformin-SGIT2I versus metformin-sulphonylureas combination therapies. The studies followed-up 1,628 participants for coronary artery disease (CAD) and acute myocardial infarction (AMI). After 2 years follow-up, there was no significant difference observed in CAD (RR = 3.01, 95% CI [0.31, 28.92], p = 0.34) and AMI (RR = 0.63, 95% CI [0.08, 5.09], p = 0.66) between the two arms. One of the two trials^57^ further evaluated 814 patients for coronary artery occlusion (CAO) and aortic aneurism and reported no significant difference in the risk of developing these diseases between the two arms (S3).

**Figure 4:**
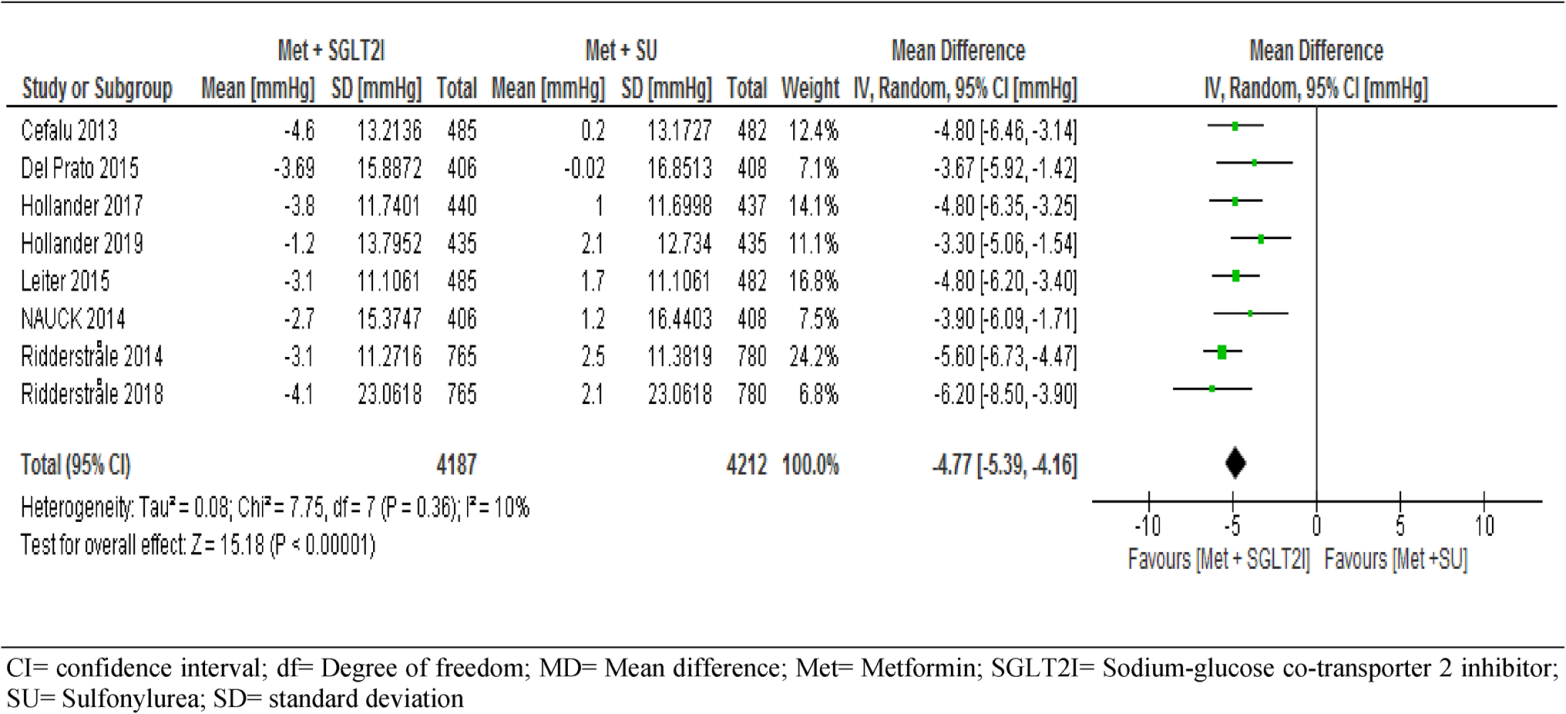
Comparison of changes in systolic blood pressure between patients who were on metformin-SGIT2I and metformin-sulphonylureas combination therapies. *CI* confidence interval; *df* degree of freedom; *MD* mean difference; *Met* metformin; *SGLT2I* sodium-glucose co-transporter 2 inhibitor; *SU* sulfonylurea; *SD* standard deviation.

**Figure 5:**
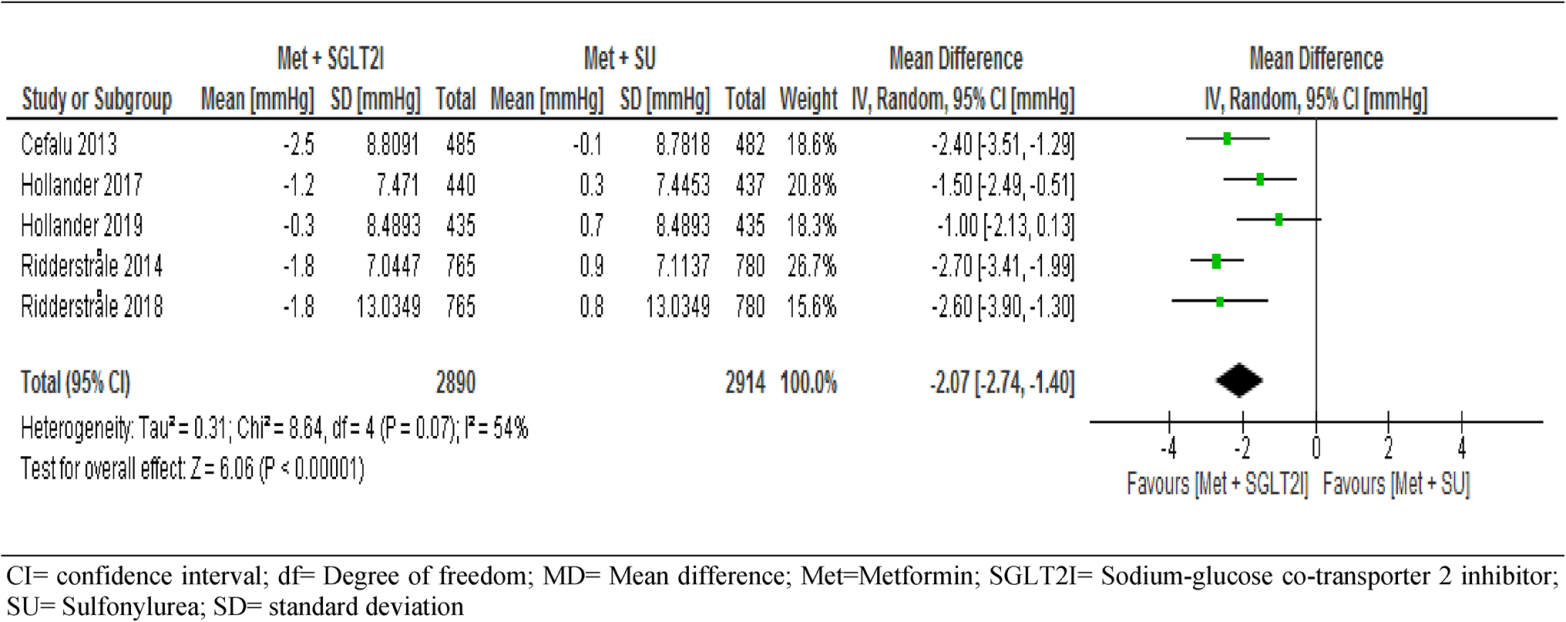
Comparison of changes in diastolic blood pressure between patients who were on metformin-SGIT2I and metformin-sulphonylureas combination therapies. *CI* confidence interval; *df* degree of freedom; *MD* mean difference; *Met* metformin; *SGLT2I* sodium-glucose co-transporter 2 inhibitor; *SU* sulfonylurea; *SD* standard deviation.

Of the nine trials included, eight trials followed-up 8,399 participants for changes in SBP and five trials followed-up 5,804 participants for changes in DBP from the baseline. The report showed a significant decreases for both SBP (MD = − 4.77, 95% CI [− 5.39, − 4.16] mmHg, p < 0.001) and DBP (MD = − 2.07, 95% CI [− 2.74, − 1.40] mmHg, p < 0.001) in patients taking metformin-SGIT2I combination therapy (Figs. 4 and 5).

Two trials^51,56^ assessed the change in HDL-C and LDL-C levels from baseline between the two arms. Both HDL-C and LDL-C levels were reduced in patients who received metformin-sulphonylureas combination therapies. However, the pooled effect was not statistically significant between the two arms in HDL-C (MD = 4.32, 95% CI [− 4.00, 12.64] mmol/L, p = 0.32), I^2^ = 99%) and LDL-C (MD = 3.63, 95% CI [− 3.96, 11.22] mmol/L, P = 0.35, I^2^ = 88%) (S14 & S15) respectively.

#### Adverse events

**Figure 6:**
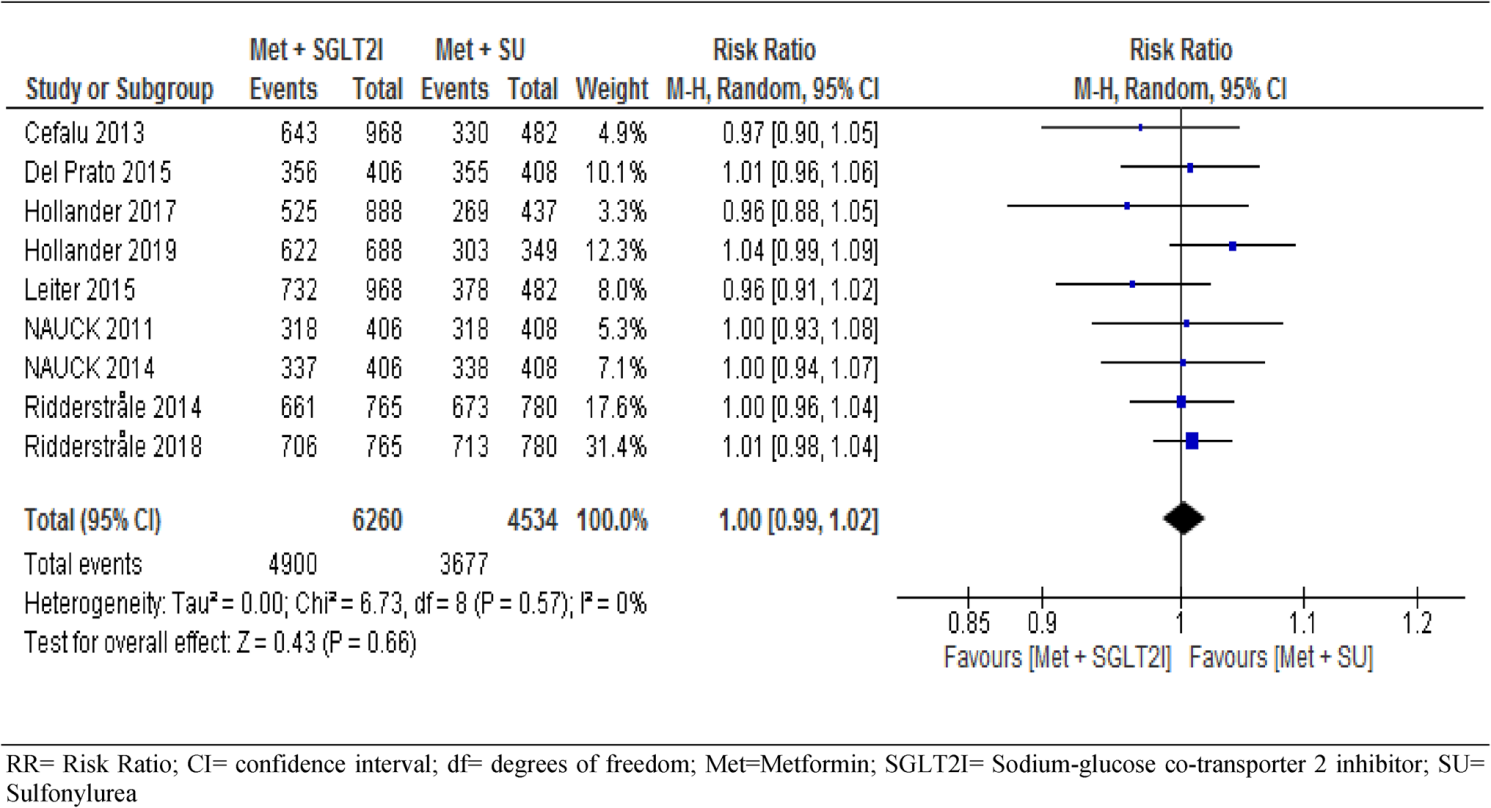
Risk of adverse events between patients who were on metformin-SGIT2I and metformin-sulphonylureas combination therapies. *RR* risk ratio; *CI* confidence interval; *df* degrees of freedom; *Met* metformin; *SGLT2I* sodium-glucose co-transporter 2 inhibitor; *SU* sulfonylurea.

With pooled data from the nine trials, we found no statistically significant difference in the risk of developing adverse events between the two arms (RR = 1.00, 95% CI [0.99, 1.02], p = 0.66, I^2^ = 0% (Fig. 6). We performed a sensitivity analysis by removing the highest weighted study^52^ and found no significant difference in the risk of developing adverse events between the two arms (RR = 1.00, 95% CI [0.98, 1.02], I^2^ = 0%). The result of the subgroup analysis was consistent at different duration of follow-up (RR = 0.98, 95% CI [0.94, 1.03] at 1 year, RR = 1.00, 95% CI [0.97, 1.04] at 2 years and RR = 1.01, 95% CI [0.98, 1.04] at 4 years, with p = 0.57 for subgroup difference (S4). Seven RCTs assessed the risk of adverse events related to study drug, of which two RCTs showed lower risk in patients on metformin-sulphonylureas combination therapies but the pooled analysis showed no statistically significant difference (RR = 0.97, 95% CI [0.85, 1.10], p = 0.60, I^2^ = 69%, (S5). Subgroup analysis of the seven RCTs showed that risk of adverse events related to study drug was similar across different duration of the study, RR = 1.09, 95% CI [0.94, 1.26] at year one, RR = 0.94, 95% CI [0.75, 1.21] at year two, and RR = 0.89, 95% CI [0.73, 1.10] at year four.

#### Serious adverse events

**Figure 7:**
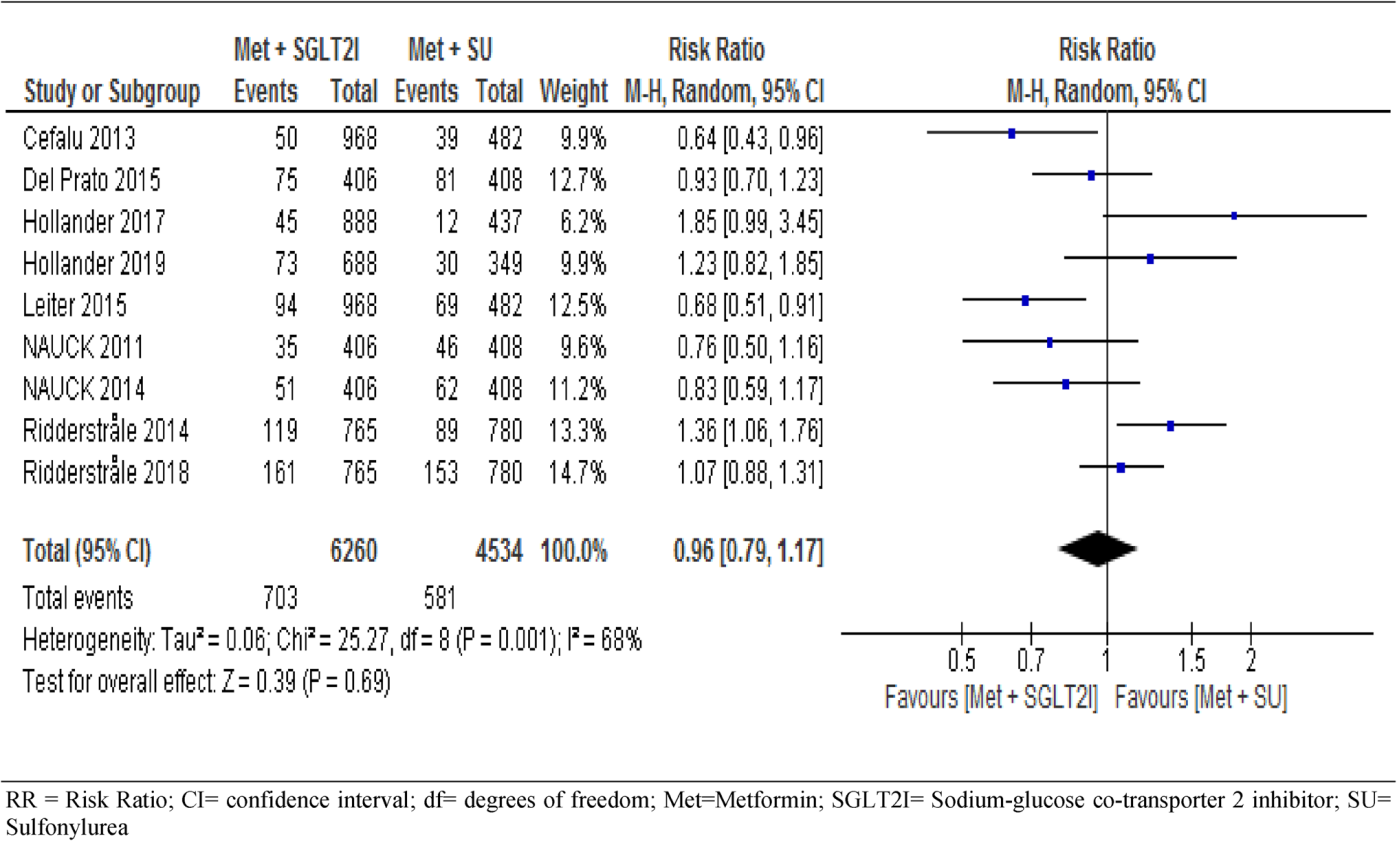
Risk of serious adverse events between patients who were on metformin-SGIT2I and metformin-sulphonylureas combination therapies. *RR* risk ratio; *CI* confidence interval; *df* degrees of freedom; *Met* metformin; *SGLT2I* sodium-glucose co-transporter 2 inhibitor; *SU* sulfonylurea.

All included trials assessed the risk of serious adverse events during the study period, where our analysis of the pooled data showed no significant difference between the two groups (RR = 0.96, 95% CI [0.79, 1.17], with statistically significant heterogeneity across trials (I^2^ = 68%, p = 0.001) (Fig. 7). Consistently, subgroup analysis showed no significant difference between the two groups at year 1 (95% CI (RR = 0.92 [0.53, 1.59], I^2^ = 75%), year 2 (RR = 0.98 [0.69, 1.40], I^2^ = 80%), and year four (RR = 1.02 [0.87, 1.20], I^2^ = 0%), with subgroup differences of p = 0.92 and I^2^ = 0% (S6).

Five trials assessed the risk of serious adverse events related to study drug. The pooled result of these trials showed that serious adverse events related to study drug were less frequent on patients taking metformin-sulphonylureas combination, but the pooled result was not significant at 95% CI (RR = 1.34 [0.75, 2.36] (S7).

#### Hypoglycemic events

**Figure 8:**
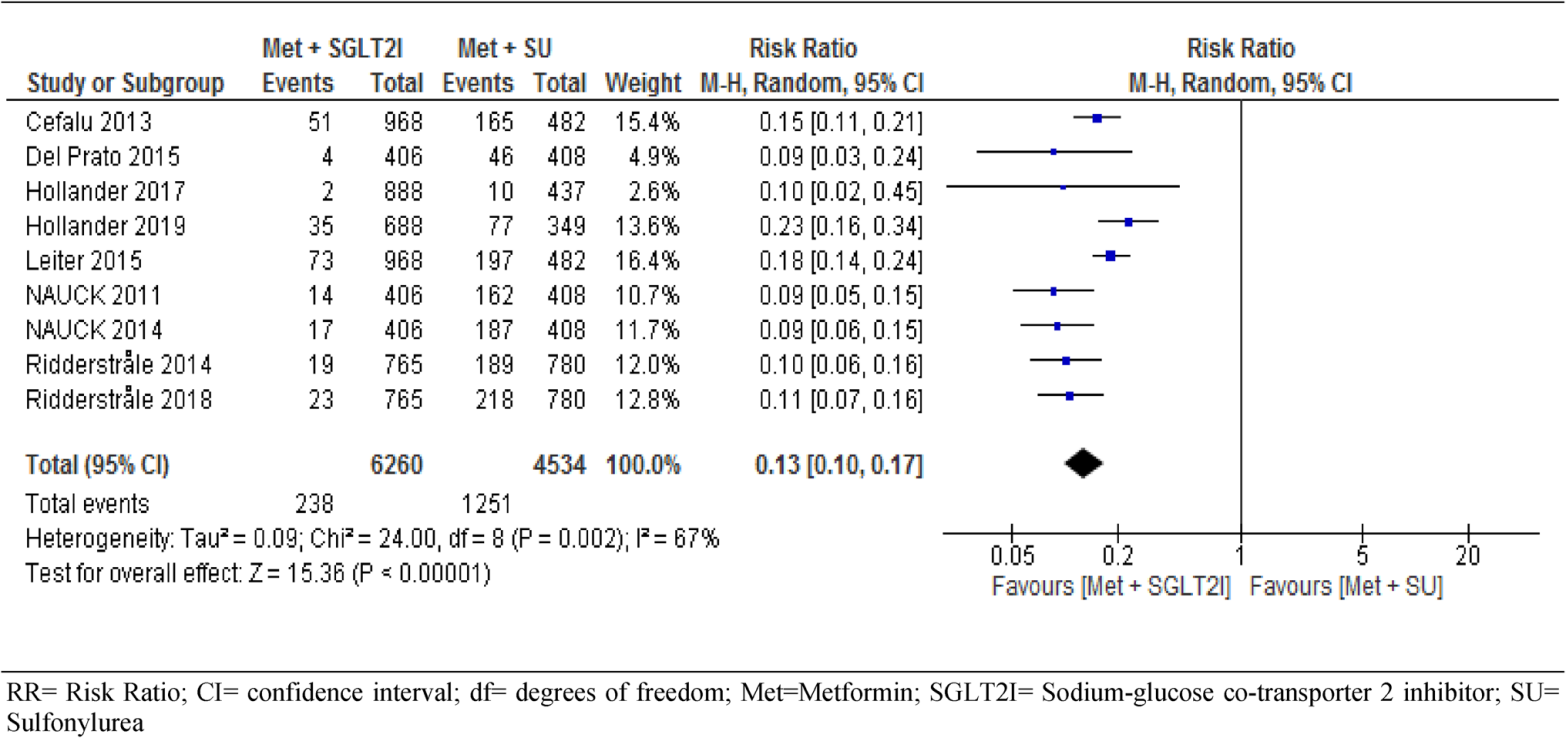
Risk of hypoglycemia between patients who were on metformin-SGIT2I and metformin-sulphonylureas combination therapies. *RR* risk ratio; *CI* confidence interval; *df* degrees of freedom; *Met* metformin; *SGLT2I* sodium-glucose co-transporter 2 inhibitor; *SU* sulfonylurea.

We analyzed hypoglycemic events on pooled results of the nine trials involving 10,794 T2DM patients, considering the occurrence of at least one hypoglycemic event during the follow-up period. Patients under metformin-SGIT2I combination therapy were found to experience significantly fewer hypoglycemic events as compared to patients under metformin-sulphonylureas combination therapy (RR = 0.13, 95% CI [0.10, 0.17], P < 0.001) I^2^ = 67%, p = 0.002) (Fig. 8). We performed sensitivity analysis by removing two low weighted trials^58,59^. However, the result of the remaining trials was similar to the nine trials in risk of hypoglycemia (RR = 0.13, 95% CI [0.10, 0.18, P < 0.001) with increased between study heterogeneity (I^2^ = 73%, P = 0.009). To see the robustness of the result, we did subgroup analysis at different duration of follow-up. However, the risk of experiencing hypoglycemic events was consistently more frequent under patients on metformin-sulfonylureas combination therapy at 95% CI RR = 0.12[0.08, 0.19], p < 0.001 at year one, RR = 0.15[0.10, 0.22], p < 0.001 at year two and RR = 0.10[0.07, 0.15], p < 0.001 at year four respectively. On the other hand, patients on metformin-SGLT2I were found to experience significantly higher GMI than patients on metformin-sulfonylureas combination therapy RR = 5.00, 95% CI [3.94, 6.33], p < 0.001 (S8).

#### Glycated hemoglobin A1c

**Figure 9:**
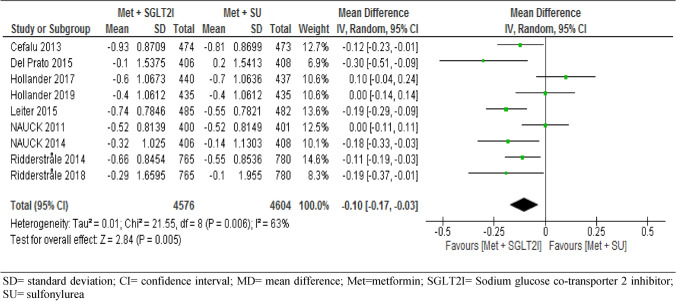
Comparison of change in HbA1c (%) from baseline between patients who were on metformin-SGIT2I and metformin-sulphonylureas combination therapies. *SD* standard deviation; *CI* confidence interval; *MD* mean difference; *Met* metformin; *SGLT2I* sodium glucose co-transporter 2 inhibitor; *SU* sulfonylurea.

All the nine RCTs involving 9,180 participants assessed the changes in HbA1c (%) from baseline between the two arms. The pooled data of these trials showed significant difference in the mean difference of HbA1c between patients on metformin-SGLT2I and metformin-sulfonylureas combination therapies; MD = − 0.10, 95% CI [− 0.17, − 0.03] %, p = 0.005), I^2^ = 63%, p = 0.006) (Fig. 9). Interestingly, subgroup analysis by duration of follow-up showed a reduction of HbA1c from baseline was not statistically significant between the two groups at year-one, with MD = − 0.01[− 0.13, 0.11] %, I^2^ = 67%. However, metformin-SGLT2I induced a greater reduction in HbA1c from baseline after 2 years (MD = − 0.12[− 0.20, − 0.05] %, p = 0.001) and after 4 years (MD = − 0.24[− 0.37, − 0.10] %, p = 0.0007) (S9).

#### Bodyweight

**Figure 10:**
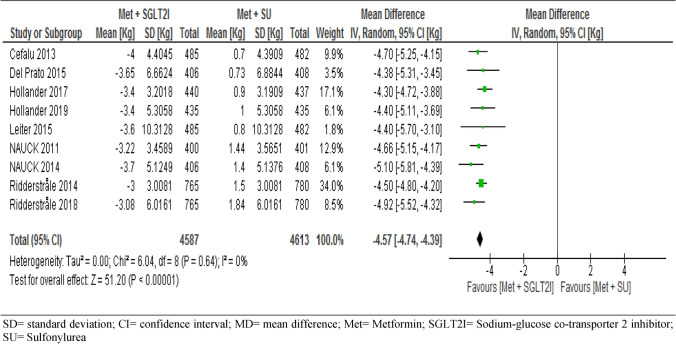
Comparison of change in body weight (Kg) from baseline between patients who were on metformin-SGIT2I and metformin-sulphonylureas combination therapies. *SD* standard deviation; *CI* confidence interval; MD = mean difference; *Met* metformin; *SGLT2I* sodium-glucose co-transporter 2 inhibitor; *SU* sulfonylurea.

All trials included in the meta-analysis assessed the change in body weight from baseline between the two groups. The pooled result showed that body weight of patients in the metformin-SGLT2I was significantly reduced from baseline compared to patients in the metformin-sulfonylureas (MD = − 4.57, 95% CI [− 4.74, − 4.39] kg, p < 0.001) (Fig. 10). We conducted a sensitivity analysis by removing the low weighted^56^ and the high weighted^53^ study and the result was consistent with the nine studies. Surprisingly, subgroup analysis also showed consistent results following different duration of follow-up that the mean difference of change in body weight from baseline at year one (MD = − 4.52 [− 4.79, − 4.24] kg, p < 0.001, at year two (MD = − 4.56 [− 4.81, − 4.31] kg, p < 0.001, and at year four (MD = − 4.76[− 5.27, − 4.26] kg, p < 0.0000, respectively (S10).

#### Fasting plasma glucose

**Figure 11:**
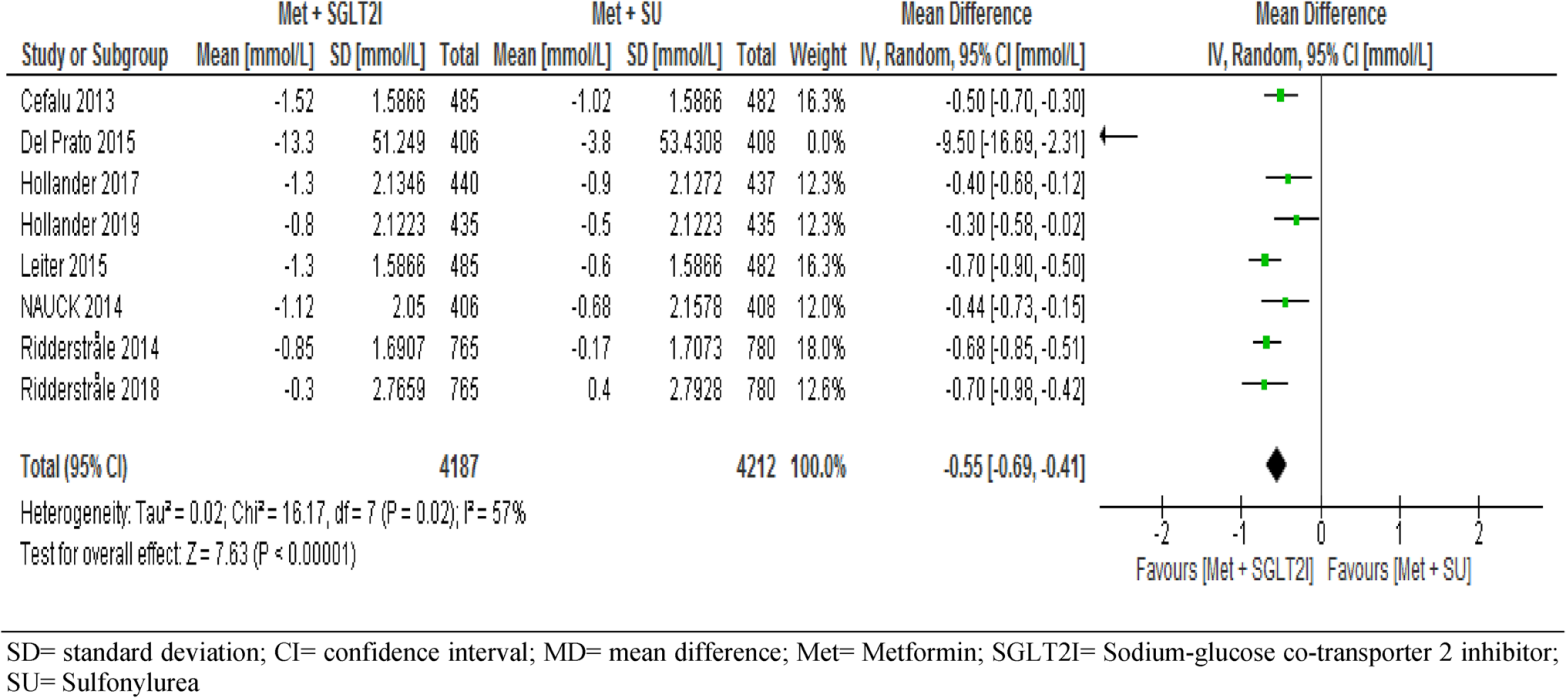
Comparison of change in FPG (mmol/L) from baseline between patients who were on metformin-SGIT2I and metformin-sulphonylureas combination therapies. *SD* standard deviation; *CI* confidence interval; *MD* mean difference; *Met* metformin; *SGLT2I* sodium-glucose co-transporter 2 inhibitor; *SU* sulfonylurea.

Eight trials assessed the change in FPG level from baseline between the intervention and control. The pooled result showed that FPG level was significantly reduced from baseline with patients in the metformin-SGLT2I combination therapies (MD = − 0.55, 95% CI [− 0.69, − 0.41] mmol/L, p < 0.001, I^2^ = 57%) (Fig. 11). We conducted sensitivity analysis by removing one outlier with a low weighted study^58^. But the result was consistent with the eight study (MD = − 0.55, 95% CI [− 0.67, − 044], p < 0.001). Subgroup analysis of seven trials also showed consistent results following different duration of intervention and control that the mean difference of change in FPG level from baseline at year one (MD = − 0.47 [− 0.63, − 0.30] mmol/L, p < 0.001, I^2^ = 0%), at year two (MD = − 0.56 [− 0.74, − 0.38] mmol/L, p < 0.001, I^2^ = 59% and at year four (MD = − 0.70[− 0.98, − 0.42] mmol/L, p < 0.001) (S11).

## Discussion

The result of this systematic review and meta-analysis showed that the risk of all-cause mortality/death events was not statistically significant between patients in the metformin-SGLT2I and metformin-sulfonylureas combination therapies. In agreement with our findings, a previous study reported a non-significant difference in all-cause mortality and cardiovascular death between the two arms^40^. Similarly, study^77^ also revealed a lower and statistically nonsignificant cardiovascular death and all-cause mortality in T2DM patients who were on metformin-sulfonylurea combination therapy. However, another study^29^ reported that the use of sulfonylureas as a second-line drug has significantly associated with an increased risk of myocardial infarction and all-cause mortality.

The goal of durable glycemic control is to reduce the long-term risk of diabetes-related cardiovascular morbidity and mortality^57^. Recent studies showed that SGLT2Is have been shown to decrease cardiovascular events in treating high-risk patients with T2DM^34^. Likewise, the addition of empagliflozin to metformin therapy improves in patients with established CVD or heart failure^78^. Even though the current meta-analysis of metformin-SGLT2I combination showed a favorable effect on cardiovascular outcomes (acute myocardial infarction, aortic aneurism, coronary artery occlusion, and atherosclerosis), the pooled analysis was not statistically significant.

Lowering blood pressure is significantly important to reduce the risk of CVD and death in many patients with T2DM. A 10-mmHg reduction in systolic blood pressure decreased the risk of major CVD events by 20%^79^. SGLT2Is induced a greater reduction in systolic and diastolic blood pressure^73^. Similar with the other findings, the pooled result of our study showed a reduction in both systolic and diastolic blood pressure with patients in the metformin-SGLT2Is combination therapy as compared with metformin-sulfonylureas, which might be due to the effect of SGLT2Is reducing renal glucose reabsorption and increasing urinary excretion^51^. Consistent with our findings, previous studies reported a greater increase from baseline in HDL-C and LDL-C in patients who were on SGLT2Is groups as compared with sulfonylureas^51,54,56^.

The result of this meta-analysis showed that the overall incidence of AEs was similar across the two arms, which is consistent with a trial reported by elsewhere^59^. However, metformin-SGLT2Is combination was associated with a higher episode of GMI which is similar to other meta-analysis reported an increased risk of GMI with SGLT2Is^73^. In addition, for both genders, the proportion of patients reporting symptoms of GMI was higher in the SGLT2Is group than in the sulfonylureas^58^. However, these infections respond to standard antimicrobial treatment and their incidence declines with time^57^. SAEs such as ketoacidosis, bone fractures, and pyelonephritis were rarely reported with metformin-SGLT2Is^53^, while the pooled result of the current study did not find a statistically significant difference between metformin-SGLT2I and metformin-sulfonylureas combinations. Even though the pooled analysis did not find statistically significant results, AEs related to study drug was observed more in patients with metformin-SGLT2Is, but more SAEs related to study drug in metformin-sulfonylureas. This might be due to the hypoglycemic effect of sulfonylureas and GMI as a result of SGLT2Is respectively.

Sulfonylureas as second-line drugs are associated with an increased risk of severe hypoglycemia^79^, and our study is in support of this. The hypoglycemic effect of sulfonylureas might be due to its insulin-dependent mechanism of action, while the less hypoglycemic effect of SGLT2Is is due to the non-insulin dependent mechanism of action.

Obesity is one of the main risk factors for T2DM and representing a major worldwide health problem. Lowering body weight is an important part of T2DM management. SGLT2Is has been associated with an added benefit of weight loss in patients with T2DM, whereas sulfonylureas are reported to increase body weight^80^. In support of this evidence, our study showed a 4.57 kg weight loss in metformin-SGLT2Is group than the metformin-sulfonylureas. The weight loss caused by SGLT2Is is probably due to the loss of calories via urine and glucose-induced osmotic diuresis^51^.

Long term glycemic control is the major goal of diabetes management to prevent both microvascular and macrovascular complications of DM^81^. Both metformin-SGLT2Is and metformin-sulfonylureas combinations are effective to control HbA1c for a short duration of follow-up. However, for a long duration of follow-up, metformin-SGLT2Is are more effective than metformin-sulfonylureas^73^. Our finding is consistent with this evidence where both groups were equally effective for a one-year duration of follow-up; however, as the duration of follow-up increases to 4 years, metformin-SGLT2Is combination showed a significant reduction from baseline in HbA1c. Eight previously conducted trials reported a higher reduction of FPG in patients randomized to metformin-SGLT2Is^51–54,56–59^, and our pooled result is in support of their finding as it showed reduction of FPG under patients on metformin-SGLT2Is.

This study reported important information about cardiovascular safety, efficacy, and cardiovascular risk factors control between the two combination therapies. However, we acknowledge that available studies are very limited and heterogeneous. There remains a need for additional long-term trials comparing the overall safety, efficacy, and cost-effectiveness of metformin-SGLT2Is and metformin-sulfonylureas combination therapies.

## Conclusion

Combination therapy of metformin and sodium-glucose cotransporter-2 inhibitors are a safe and efficacious alternative to combination therapy of metformin and sulfonylureas for patients with T2DM who are at risk of cardiovascular comorbidity. However, there remains a need for additional long-term randomized controlled trials as available studies are very limited and heterogeneous.

## Supplementary Information

Supplementary Information 1.

Supplementary Information 2.

## Data Availability

All relevant data are within the manuscript and its supporting information files.

## Abbreviations

AEs: Adverse events
AMI: Acute myocardial infraction
CAD: Coronary artery disease
CAO: Coronary artery occlusion
CVD: Cardiovascular disease
DBP: Diastolic blood pressure
FPG: Fasting plasma glucose
GMI: Genital mycotic infection
HbA1C: Hemoglobin A1c
HDL-C: High-density lipoprotein cholesterol
LDL-C: Low-density lipoprotein cholesterol
MD: Mean difference
MI: Myocardial infraction
RCT: Randomized controlled trials
RR: Risk Ratio
SAEs: Serious adverse events
SBP: Systolic blood pressure
SGLT2Is: Sodium-glucose Co-transporter 2 inhibitors
T2DM: Type 2 diabetes mellitus
