# Supplementary material for: Cardiovascular safety and efficacy of metformin-SGLT2i versus metformin-sulfonylureas in type 2 diabetes: systematic review and meta-analysis of randomized controlled trials
Source: Sci Rep. 2021 Jan 8;11:137. doi: 10.1038/s41598-020-80603-8 (PMC7794474; doi:10.1038/s41598-020-80603-8)
Supplement: Supplementary file 2 — Supplementary Information 2. [file 41598_2020_80603_MOESM2_ESM.docx]

**Cardiovascular safety and efficacy of metformin-SGLT2i versus metformin-sulfonylureas in type 2 diabetes: systematic review and meta-analysis of randomized controlled trials**

Desye Gebrie*^1, 2^, Desalegn Getnet^3^, Tsegahun Manyazewal^2^

^1^School of Pharmacy, College of Health Sciences, Mekelle University, Mekelle, Ethiopia

^2^Addis Ababa University, College of Health Sciences, Center for Innovative Drug Development and Therapeutic Trials for Africa (CDT-Africa), Addis Ababa, Ethiopia

^3^Pharmacology and Toxicology Course and Research Team, Department of Pharmacy, College of Health Sciences, Adigrat University, Adigrat, Ethiopia

Correspondence: Desye Gebrie: [desye.gebrie@mu.edu.et](mailto:desye.gebrie@mu.edu.et). ^1^School of Pharmacy, College of Health Sciences, Mekelle University, Mekelle, Ethiopia; ^2^Addis Ababa University, College of Health Sciences, Center for Innovative Drug Development and Therapeutic Trials for Africa (CDT-Africa), Addis Ababa, Ethiopia, P.O. Box 9086.

Co-authors’ Email Address

Desalegn Getnet: [desget361@gmail.com](mailto:desget361@gmail.com)

Tsegahun Manyazewal: [tsegahunm@gmail.com](mailto:tsegahunm@gmail.com)

# Supplementary files (S1-S15)

**S1**: PubMed search strategies

| # | **Searches** | **Results** |
| --- | --- | --- |
| 1 | "metformin"[MeSH Terms] OR "metformin"[All Fields] OR "metformine"[All Fields] OR "metformin s"[All Fields] OR "metformins"[All Fields] OR "biguanid"[All Fields] OR "biguanides"[MeSH Terms] OR "biguanides"[All Fields] OR "biguanide"[All Fields] OR "biguanids"[All Fields] | 36,949 |
| 2 | ("sodium-glucose"[All Fields] AND "co transporter"[All Fields] AND "2"[All Fields] AND ("antagonists and inhibitors"[MeSH Subheading] OR ("antagonists"[All Fields] AND "inhibitors"[All Fields]) OR "antagonists and inhibitors"[All Fields] OR "inhibitors"[All Fields] OR "inhibitor"[All Fields] OR "inhibitor s"[All Fields])) OR ("sodium glucose transporter 2 inhibitors"[Pharmacological Action] OR "sodium glucose transporter 2 inhibitors"[MeSH Terms] OR "sodium glucose transporter 2 inhibitors"[All Fields] OR "sglt 2 inhibitors"[All Fields]) OR ("sodium glucose transporter 2 inhibitors"[Pharmacological Action] OR "sodium glucose transporter 2 inhibitors"[MeSH Terms] OR "sodium glucose transporter 2 inhibitors"[All Fields] OR ("sglt2"[All Fields] AND "inhibitors"[All Fields]) OR "sglt2 inhibitors"[All Fields]) OR ("2 3 4 ethoxybenzyl 4 chlorophenyl 6 hydroxymethyltetrahydro 2h pyran 3 4 5 triol"[Supplementary Concept] OR "2 3 4 ethoxybenzyl 4 chlorophenyl 6 hydroxymethyltetrahydro 2h pyran 3 4 5 triol"[All Fields] OR "dapagliflozin"[All Fields]) OR ("canagliflozin"[MeSH Terms] OR "canagliflozin"[All Fields]) OR ("empagliflozin"[Supplementary Concept] OR "empagliflozin"[All Fields]) OR ("ertugliflozin"[Supplementary Concept] OR "ertugliflozin"[All Fields]) | 5,266 |
| 3 | 1 AND 2 | 994 |
| 4 | "sulfonylurea compounds"[MeSH Terms] OR ("sulfonylurea"[All Fields] AND "compounds"[All Fields]) OR "sulfonylurea compounds"[All Fields] OR "sulfonylurea"[All Fields] OR "sulfonylureas"[All Fields] OR "sulphonylurea"[All Fields] OR "sulphonylureas"[All Fields] OR ("gliclazide"[MeSH Terms] OR "gliclazide"[All Fields]) OR ("glimepiride"[Supplementary Concept] OR "glimepiride"[All Fields]) OR ("glibenclamid"[All Fields] OR "glyburide"[MeSH Terms] OR "glyburide"[All Fields] OR "glibenclamide"[All Fields] OR "glyburide s"[All Fields]) OR ("glibenclamid"[All Fields] OR "glyburide"[MeSH Terms] OR "glyburide"[All Fields] OR "glibenclamide"[All Fields] OR "glyburide s"[All Fields]) OR ("glipizide"[MeSH Terms] OR "glipizide"[All Fields]) OR ("tolbutamide"[MeSH Terms] OR "tolbutamide"[All Fields]) | 30,198 |
| 5 | 1 AND 4 | 5,305 |
| 6 | "diabetes mellitus, type 2"[MeSH Terms] OR "type 2 diabetes mellitus"[All Fields] OR "T2DM"[All Fields] OR (("maturate"[All Fields] OR "maturated"[All Fields] OR "maturating"[All Fields] OR "maturation"[All Fields] OR "maturational"[All Fields] OR "maturations"[All Fields] OR "maturative"[All Fields] OR "mature"[All Fields] OR "matured"[All Fields] OR "maturer"[All Fields] OR "maturers"[All Fields] OR "matures"[All Fields] OR "maturing"[All Fields] OR "maturities"[All Fields] OR "maturity"[All Fields]) AND "set"[All Fields] AND ("diabetes mellitus"[MeSH Terms] OR ("diabetes"[All Fields] AND "mellitus"[All Fields]) OR "diabetes mellitus"[All Fields])) OR ("diabetes mellitus, type 2"[MeSH Terms] OR "type 2 diabetes mellitus"[All Fields] OR ("non"[All Fields] AND "insulin"[All Fields] AND "dependent"[All Fields] AND "diabetes"[All Fields] AND "mellitus"[All Fields]) OR "non insulin dependent diabetes mellitus"[All Fields]) | 157,378 |
| 7 | (("cardiovascular system"[MeSH Terms] OR ("cardiovascular"[All Fields] AND "system"[All Fields]) OR "cardiovascular system"[All Fields] OR "cardiovascular"[All Fields] OR "cardiovasculars"[All Fields]) AND ("outcome"[All Fields] OR "outcomes"[All Fields])) OR ("cardiovascular diseases"[MeSH Terms] OR ("cardiovascular"[All Fields] AND "diseases"[All Fields]) OR "cardiovascular diseases"[All Fields] OR ("cardiovascular"[All Fields] AND "disease"[All Fields]) OR "cardiovascular disease"[All Fields]) OR "CVD"[All Fields] | 2,613,638 |
| 8 | "randomized controlled trial"[Publication Type] OR "randomized controlled trials as topic"[MeSH Terms] OR "randomized controlled trials"[All Fields] OR "randomised controlled trials"[All Fields] OR "RCRs"[All Fields] OR "clinical trial"[Publication Type] OR "clinical trials as topic"[MeSH Terms] OR "clinical trials"[All Fields] | 1,350,235 |
| 9 | 6 AND 7 AND 8 | 8,673 |
| 10 | 3 AND 5 AND 9 | 1463 |

**S2**: Subgroup analysis of death events between patients who were on metformin-SGLT2I and metformin-sulfonylureas combination therapies by duration of treatment.

**
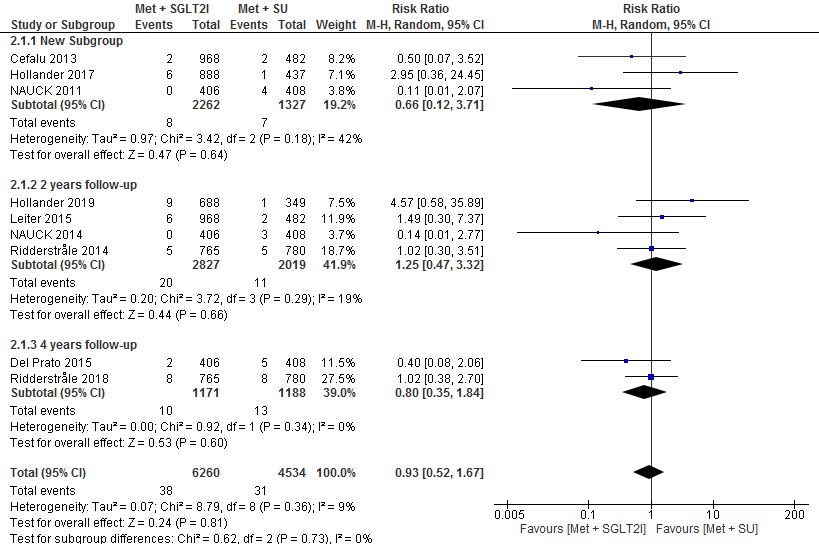
**

**S3**: Comparison of cardiovascular events between patients who were on metformin-SGLT2I and metformin-sulfonylureas combination therapies

1.
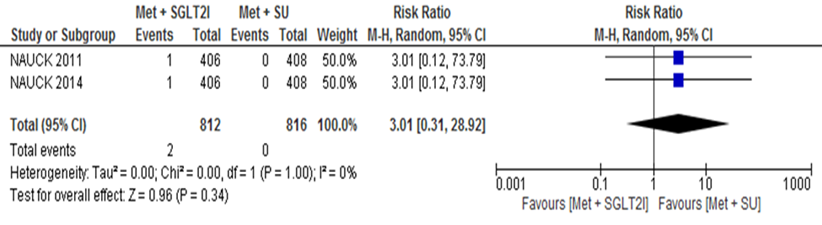

2.
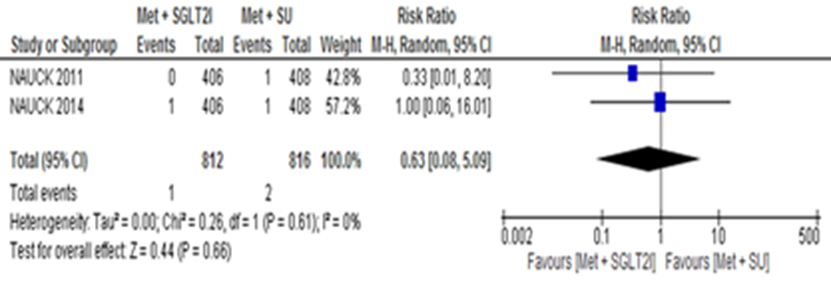

3.
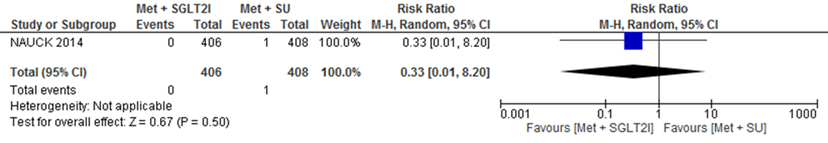

4.
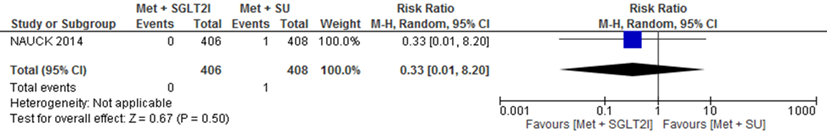


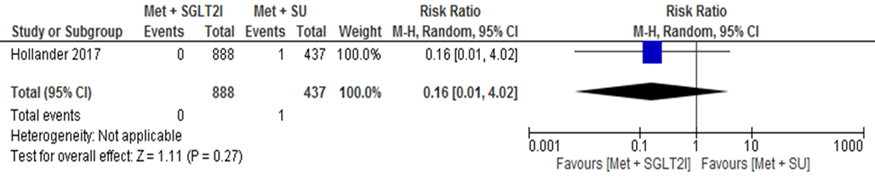


^A: Worsening of coronary artery disease; B: Acute myocardial infraction; C: Coronary artery occlusion; D: Aortic aneurism; E: Atherosclerosis^

**S4:** Subgroup analysis of adverse event between patients who were on metformin-SGLT2I and metformin-sulfonylureas combination therapies by durationof treatment.

**
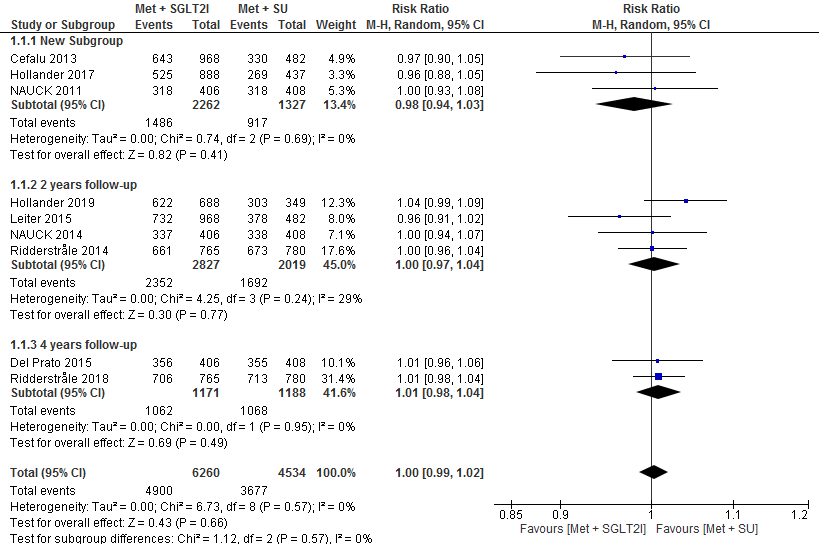
**

**S5:** Comparison of adverse events related to study drug between patients who were on metformin-SGLT2I and metformin-sulfonylureas combination therapies.


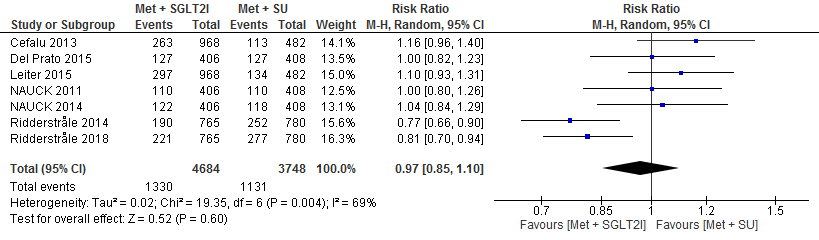


**S6:** Subgroup analysis of serious adverse event between patients who were on metformin-SGLT2I and metformin-sulfonylureas combination therapies by duration of treatment.

**
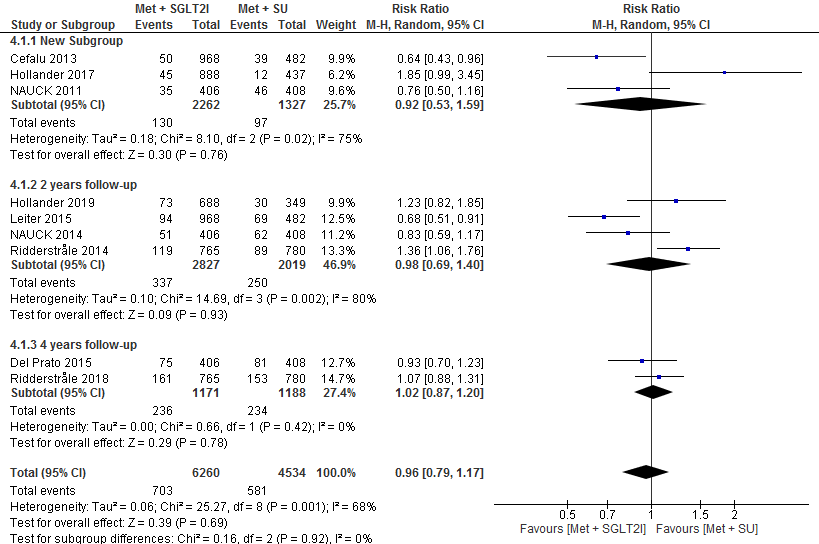
**

**S7:** Comparison of serious adverse events related to study drug between patients who were on metformin-SGLT2I and metformin-sulfonylureas combination therapies.


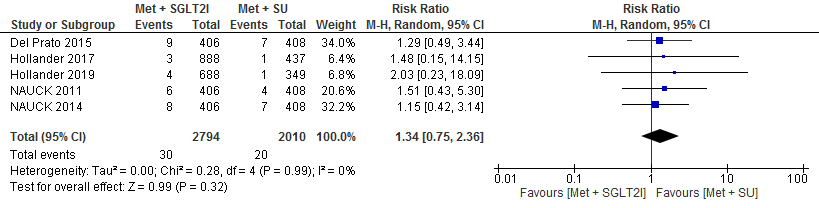


**S8:** Subgroup analysis of hypoglycemic event between patients who were on metformin-SGLT2I and metformin-sulfonylureas combination therapies by duration of treatment.

**
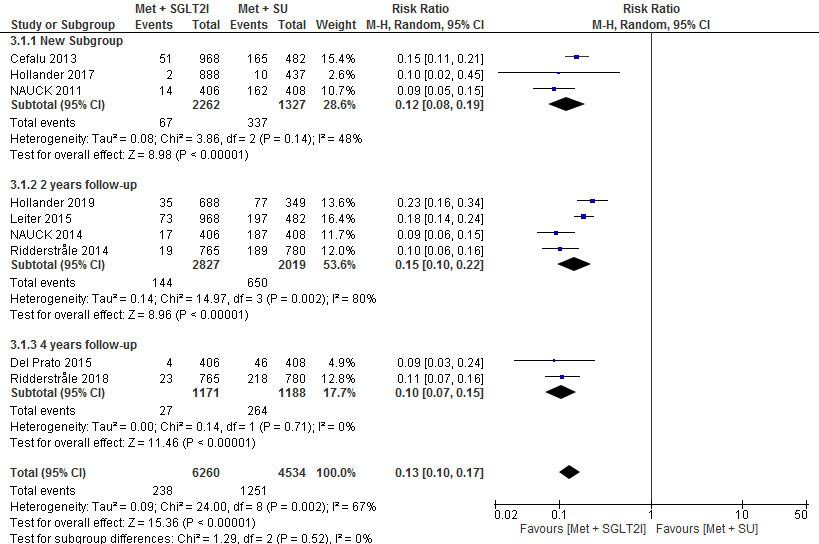
**

**S9:** Subgroup analysis of change in HbA1c (%) from baseline between patients who were on metformin-SGLT2I and metformin-sulfonylureas combination therapies by duration of treatment.

**
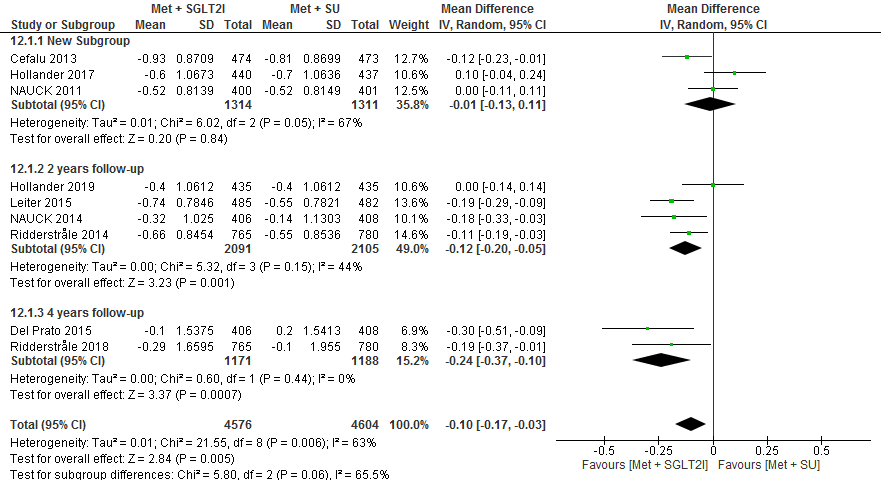
**

**S10:** Subgroup analysis of change in body weight (Kg) from baseline between patients who were on metformin-SGLT2I and metformin-sulfonylureas combination therapies by duration of treatment.

**
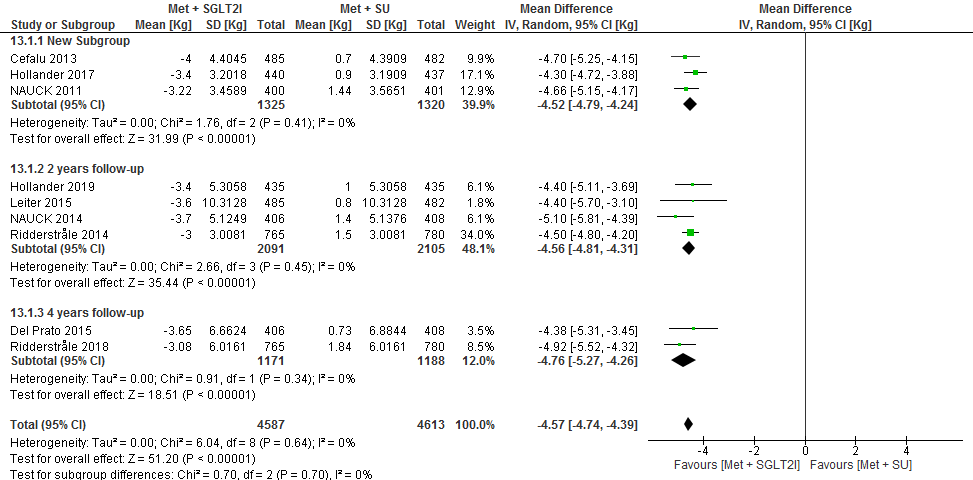
**

**S11:** Subgroup analysis of change in FPG (mmol/L) from baseline between patients who were on metformin-SGLT2I and metformin-sulfonylureas combination therapies by duration of treatment.

**
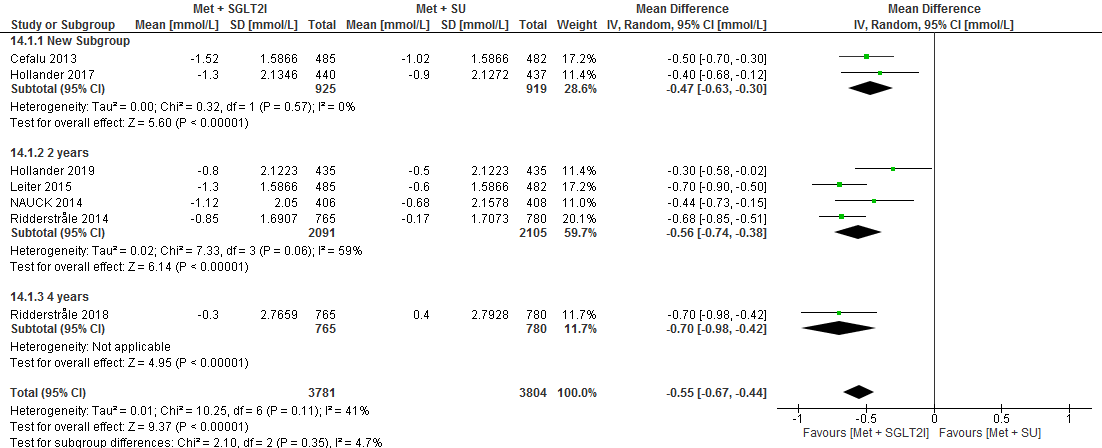
**

# S12: Sensitivity analysis for the risk of advers events by removing the highest weight study (Ridderstale, 2018)


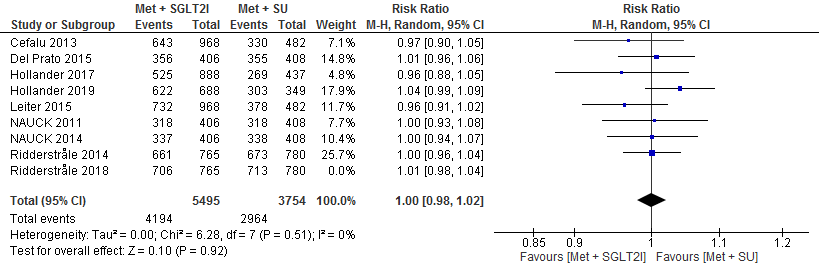


**S13:** Comparison of risk of genital mycotic infection between patients who were on metformin-SGLT2I and metformin-sulfonylureas combination therapies.


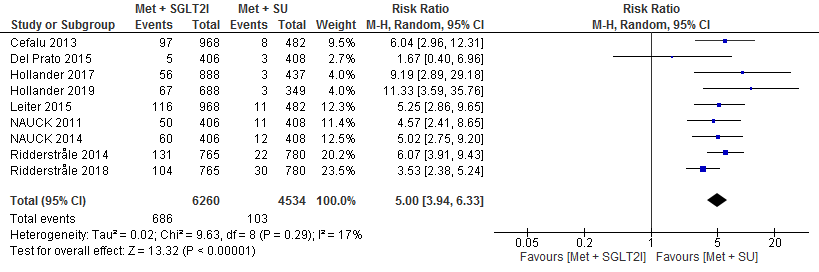


**S14:** Comparison of HDL-C between patients who were on metformin-SGLT2I and metformin-sulfonylureas combination therapies.


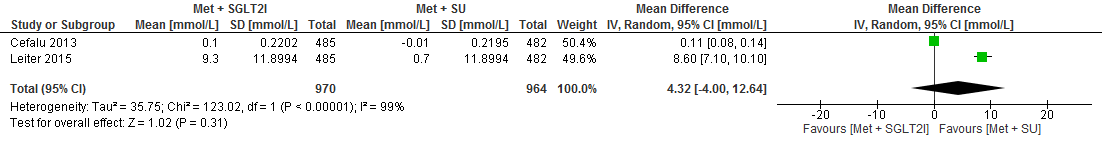


**S15:** Comparison of LDL-C between patients who were on metformin-SGLT2I and metformin-sulfonylureas combination therapies.

**
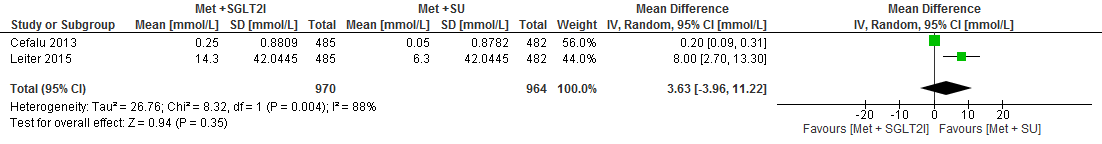
**
